# Supplementary material for: Development of a digital tool to assist in monitoring compliance for a public health initiative: “A Better Choice Food and Drink Supply Strategy for Queensland Healthcare Facilities”
Source: PLoS One. 2026 Feb 20;21(2):e0341747. doi: 10.1371/journal.pone.0341747 (PMC12922977; doi:10.1371/journal.pone.0341747)
Supplement: S1 Table — (PDF) [file pone.0341747.s001.pdf]

S1 Table. Specific targets for retail outlets and vending machines food and drinks

Category	Green	Amber	Red	Artificially sweetened drinks
**Food**				
Retail outlet	>=50%	-	<=20%	-
Vending machine	>=30%	-	<=20%	-
**Drink**				
Retail outlet	>=50%	-	0%	<=20%
Vending machine	>=50%	-	0%	<=20%
