# Supplementary material for: Development of a digital tool to assist in monitoring compliance for a public health initiative: “A Better Choice Food and Drink Supply Strategy for Queensland Healthcare Facilities”
Source: PLoS One. 2026 Feb 20;21(2):e0341747. doi: 10.1371/journal.pone.0341747 (PMC12922977; doi:10.1371/journal.pone.0341747)
Supplement: Attachment — Submitted filename: Response to Reviewers.docx [file pone.0341747.s005.docx]

**STATEMENT ADDRESSING REVIEWERS’ COMMENTS**

Note: Responses to reviewers’ comments are in blue. Changes made to the manuscript are in underlined red. Page and paragraph numbers cited herein pertain to the marked-up version provided.

**Reviewer #1:**

The work require a mix of professionals and a high level of IT involvement. A considerable work has been done.

Thank you for acknowledging the interdisciplinary nature and technical complexity of this project.
1. User interface and accessibility: (I) how user-friendly it is? Any advantage in relation to other systems used earlier? Interactivity????

Thank you for raising these important points regarding the dashboard’s usability and interactivity. As stated in the manuscript, we used Power BI to develop the dashboard which is widely accessible via desktop, web, and mobile apps and widely used across HWQld and HHSs. We designed the user interface with a strong emphasis on simplicity, clarity, and interactivity. The layout ensures that users – regardless of technical background – can intuitively navigate the dashboard. Key features such as dropdown filters, hover-over tooltips, and responsive charts allow users to explore data dynamically without requiring prior training.

Comparing to other systems such as the Australian food environment dashboard ([foodenvironmentdashboard.com.au](https://foodenvironmentdashboard.com.au/)) which reports food availability across hospital and heath services in Western Australia and New South Wales along with compliance with government policy and recommendations, our dashboard provides detailed audit information in a user-friendly and highly interactive way. Our dashboards inclusion of comprehensive data visualisation and interactivity are key design features that set it apart.

To highlight this, the advantages of user interface and interactivity have been added to the manuscript in the method and discussion sections *(paragraph 3, page 8 and paragraph 4 page 11).*

*“The dashboard was designed to be user-friendly and in a form that could potentially be deployed to HHSs to stay informed about their compliance data. Its comprehensive nature and layout prioritise intuitive navigation, enabling users, regardless of technical expertise, to engage with the dashboard effectively. Key interactive features, including dropdown filters, hover-over tooltips, and responsive charts, facilitate dynamic data exploration without the need for prior training.*”

*“We have successfully developed an interactive dashboard that is accessible to HWQld and shareable with HHSs to monitor compliance with the Directive. To date, there are no directly comparable systems that provide an accessible, policy-aligned, and visually structured overview of food environments within Australian hospitals and health services. While nutrition audit reports from jurisdictions such as Western Australia and New South Wales have offered valuable insights into compliance with healthy food policies, these reports are typically static and lack the interactive, user-oriented design featured in this dashboard (6). Compared to the traditional paper-based report, this new approach achieved faster turnaround times in providing feedback to HHSs due to streamlined survey structures and improved processes for data exporting, cleaning and analysis.”*

2. The idea compliance is weighted by service volume for each facility is not clear, because I could not see relationship between food and drink provided and compliance. The principle, I thought, is compliance regardless of the size of the institution served.
The decision to apply service volume weighting was based on the principle of equity and proportional impact. Facilities that serve a greater volume of the public and hence a larger number of meals or beverages have a greater influence on the overall food environment within the health system. Weighting compliance scores by service volume ensures that the evaluation reflects not just whether healthy options are available, but how widely they are accessed. This approach captures the real-world exposure of consumers to healthier (or less healthy) choices, giving greater analytical weight to facilities that shape dietary patterns at scale. In doing so, it supports a more equitable and representative assessment of system-wide food service performance.

For example, a small facility with high compliance may have limited reach, whereas a large hospital with low compliance could affect thousands of consumers daily. Weighting ensures that the dashboard captures this difference in scale and better represents the population-level implications of food environment policies.

That said, we acknowledge that this approach may not be immediately intuitive and recognize the value of presenting both unweighted compliance alongside weighted to allow users to interpret both perspectives. No changes were made to the manuscript.

3. In the Discussion section, authors could not compare the system with any other system which may have similar function. If this is completely new, it is good to say it loudly. Even no comparison was made with the previous system to show the relative advantage of the current one.

To our knowledge, there are no directly comparable systems that offer an accessible, policy-aligned, and visually structured overview of food environments within Australian hospitals and health services. While nutrition audit reports such as those conducted by the Western Australia Department of Health and New South Wales Health provide valuable data on compliance with healthy food policies, these are typically presented in static formats and lack the interactive, user-friendly interface offered by the Food Environment Dashboard ([foodenvironmentdashboard.com.au](https://foodenvironmentdashboard.com.au/)). We have now emphasised this advantage in the manuscript as described in response 1 (paragraph 4, page 11).

4. It would have been good to also use the data for some statistical analysis to show it on the dashboard

Statistical analysis is only useful if there is a specific question of difference that is trying to be assessed. For this project, there were no questions of difference proposed that required a statistical analysis. That said, we thank you for this suggestion and agree that incorporating statistical analysis using real audit data could strengthen the demonstration of the dashboard’s analytical capabilities when appropriate. We have added in paragraph 3, page 12 a statement to integrate statistical tools to analyse trend and identify differences in compliance rates across HHSs as one of the upcoming focus areas.

*Paragraph 4, page 12 now reads:*

*“The dashboard is currently accessible to policy makers but not available to HHSs. The current design of the dashboard allows for a number of options for sharing; however, maintaining a robust digital dashboard requires significant technical expertise and infrastructure [5]. Ensuring the privacy and security of the data is critical. Effective use of dashboards requires training for users to interpret and use the data correctly [5]. All these aspects need to be considered before sharing the dashboards to broader users. Upcoming efforts will also focus on exploring effective methods for communicating results to individual retailers and determining which entities fall within the scope of the Directive. Future enhancements to the dashboard may include integrated statistical tools to analyse trend and identify differences in compliance rates.”*

**Reviewer #2:**

Development of a digital tool to assist in monitoring compliance for a public health initiative: "A Better Choice Food and Drink Supply Strategy for Queensland Healthcare Facilities"
Thank you for the opportunity to review this manuscript. It is a timely and engaging contribution to the field, particularly in its innovative approach to visualising policy-related data on food and drink availability in Queensland. The development of a dashboard tool to support both the interpretation of results and the monitoring of compliance with the ABC Policy is a valuable initiative that uses existing platforms and software. The manuscript is generally well-structured and clearly written. Below is a series of comments that aim to support further refinement of the manuscript.

***1. Scope and Policy Context***
The manuscript appropriately focuses on Queensland, which is appropriate given the jurisdictional differences in policy targets across Australian states. A brief note acknowledging that different states have varying policies and policy targets could enhance the clarity for readers outside Australia and indicate whether the tool could be adapted for use in other jurisdictions. This would help readers understand the broader relevance of the approach.
Thank you for this thoughtful suggestion. We agree that acknowledging the variation in food and nutrition policies across Australian states would enhance clarity, particularly for international readers. In response, we have revised the Discussion section to include a brief note highlighting these jurisdictional differences and the implications for dashboard adaptability. While the current dashboard is tailored to Queensland’s Directive, its modular structure, policy-aligned metrics, and scalable design allow for adaptation to other jurisdictions with similar institutional food environment policies. This addition helps contextualize the broader relevance of the tool and its potential application beyond Queensland.

*Paragraph 2, page 13 now reads:*

*“While this dashboard has been developed specifically for Queensland, its design principles and monitoring framework have broader applicability. It is important to note that food and nutrition policies vary significantly across Australian states and territories, with each jurisdiction adopting distinct policy targets, implementation strategies, and compliance mechanisms. By aligning the dashboard with the Queensland Directive, we ensured contextual relevance; however, the underlying structure—particularly its modular design, service-volume weighting, and visual reporting tools—could be adapted to suit other jurisdictions across Australia and internationally. Acknowledging these policy differences not only clarifies the scope of the current tool but also highlights its potential as a scalable model for national or cross-jurisdictional monitoring of institutional food environments.”*
***2. Introduction***
The introduction outlines several monitoring systems, but it would be strengthened by the inclusion of more examples directly related to nutrition and food environments. Relevant dashboards include the World Obesity Observatory (<https://data.worldobesity.org/>), the GBD Compare tool (<https://vizhub.healthdata.org/gbd-compare/>), and the Australian Food Environment Dashboard (<https://foodenvironmentdashboard.com.au/>) - but others can also be included. Briefly summarising the focus of such platforms would situate the study within ongoing public health nutrition initiatives and underline the rationale for the proposed dashboard (even if only available internally). These examples also reflect a broader trend away from static, report-heavy outputs towards interactive, user-driven platforms that allow tailored exploration of data (especially for time-poor policy makers).

Thank you for this valuable suggestion. We agree that situating our dashboard within the broader landscape of public health nutrition monitoring tools will enhance the relevance and clarity of the Introduction. In response, we have revised the section to include examples of established platforms such as the Australian Food Environment Dashboard, Global Food Environment Dashboard, and Food System Dashboard. These examples illustrate the growing shift toward interactive, user-driven systems that support tailored data exploration—particularly beneficial for policy makers and practitioners. This addition helps contextualize our dashboard within ongoing global and national efforts to improve food environments and public health outcomes.

*Paragraph 3, page 4 now reads:*

*“Still, there is sparse evidence on the use of digital dashboards to monitor preventive health policies, particularly in the domain of nutrition and institutional food environments. While platforms such as the Australian Food Environment Dashboard (6), Global Food Environment Dashboard (7), Food System Dashboard (8) demonstrate the growing use of interactive tools in public health nutrition, they primarily focus on population-level indicators and broader environmental metrics. These examples reflect a broader trend away from static, report-heavy outputs toward dynamic, user-driven platforms that support tailored data exploration—especially valuable for time-poor policymakers. Building on this momentum, we aim to present a case study for developing a visual digital dashboard to monitor a preventive health policy related to nutrition within Queensland hospitals and health services”.*

3. The first paragraph on page 4 briefly outlines findings from the scoping review. However, this section would benefit from examples clarifying what "user requirements, robust infrastructure and intuitive interfaces" entail in practice. For instance, how such requirements shape dashboard design and usability.
NB Page 5 paragraph 1: There appears to be "(ref)" included after (8), possibly a typo.
Thank you for citing the typo and also providing this helpful suggestion. We agree that providing concrete examples of how user requirements, infrastructure, and interface design influence dashboard development would enhance clarity and relevance. In response, we have revised the paragraph to include practical illustrations drawn from the scoping review and our own dashboard experience. These additions help demonstrate how such considerations translate into design decisions that improve usability, accessibility, and functionality for end users.

*Paragraph 1 page 4 now reads:*

*“Current literature highlights the significant role of digital health dashboards in the context of public health risks and diseases. A systematic review examining the state of research on dashboards in the context of public health risks and diseases found 65 studies reported the development of digital dashboards to monitor infectious diseases (e.g., Dengue, Corona, Ebola; N=21), crises (e.g., disaster, emergencies; N=10), health related services (e.g., aging population, health promotion programs; N=17), and other health hazards (e.g., substance abuse, pollution; N=17) [4]. The systematic review noted that while many studies focused on the functional aspects of dashboards, there was a need for more user-centric evaluations [4].* [*A scoping review published in 2024 identified key design principles for developing effective public health surveillance dashboards, emphasizing the importance of user requirements, robust infrastructure, and intuitive interfaces*](https://bmcpublichealth.biomedcentral.com/articles/10.1186/s12889-024-17841-2) *[5]. Examples of these elements include user requirements such as fast data access, customizable views, and minimal training shape dashboard design through filterable visuals, simple navigation, and role-specific access. Robust infrastructure ensures secure handling of large datasets and timely updates via scalable systems and reliable data pipelines. Intuitive interfaces reduce cognitive load with clean layouts, consistent icons, and interactive features like tooltips and drilldowns.”*
4. **Clarifications**
The manuscript refers to traffic light food and drink categories. On page 5, paragraph 2, 'red' items should it say (limited or no nutritional value)? It would be helpful to include a brief explanation of what specific targets or thresholds apply under the policy to the green, amber and red categories, and why "artificially sweetened beverages" are treated differently as a separate category (and what is the target for these)? This would improve accessibility for readers unfamiliar with the policy framework.

Thank you for this helpful suggestion. We agree that clarifying the traffic light classification system and associated targets will improve accessibility for readers unfamiliar with the policy. In response, we have revised the manuscript to define the green, amber, and red categories more explicitly, including the nutritional rationale behind each classification. We have also added a brief explanation of the specific thresholds applied to each category under the policy and clarified why artificially sweetened beverages are treated as a separate category, including the volume restriction that applies (Table S1). These additions aim to enhance clarity and contextual understanding for a broader audience.

*Paragraph 3, page 5 now reads:*

*“ABC uses a traffic light system, based on the Australian Dietary Guidelines, to classify food and drinks as green (best nutritional value), amber (some nutritional value), or red (limit or no nutritional value) [10]. The Directive sets specific targets for retail outlets and vending machines (see Table S1): for example, for food in retail outlet at least 50% of items must be green, no more than 20% may be red, and the remainder may be amber. Artificially sweetened (AS) beverages, such as diet soft drinks, are treated as a separate category due to their low energy content but potential influence on taste preferences and consumption behaviours. The specific target for AS drink is not more than 20%.”*

5. The term "Mater Health" is used without explanation. A brief clarification in brackets or a footnote would be helpful for readers unfamiliar with Queensland's health system structure.

Mater Health is a publicly funded hospital operating under its own act and is different to HHSs. This is now clarified in the manuscript.

*Paragraph 2, page 6 now reads:*

*“Queensland has 16 HHSs (geographically managed health systems that are groups of hospitals under unified management) and Mater Health (a publicly funded hospital operating under its own act), which collectively provide public hospital and health services to a population of 5,215,000 people. In compliance with the Directive, Health providers (HHSs) are responsible for auditing the food and drinks supplied in retail outlets and vending machines within their facilities on an annual basis, which include a mix of charity, commercial and hospital operated outlets. This data is then utilised to assess adherence to the Directive. HWQld is required to perform subsequent analysis, report compliance to the Queensland Department of Health and provide feedback to each HHS on their performance and plans for improvement.*”

6. Please include the ref number for the ethics approval.
The ref number for the ethics approval has been added.

*Paragraph 2, page 7 now reads:*

*“The study was approved by the University of Queensland Human Research Ethics Committee (Ref: 2023/HE002184).”*
**7. Purpose of the Dashboard and Aim of the Study**
The manuscript states that the purpose of the dashboard is to monitor compliance/trends. However, it is more of a visualising tool for results and trends since the monitoring includes more broadly all actions taken to obtain the results (which does not diminish its value). The authors should use the terminology more consistently across the different parts of the manuscript.

Thank you for this thoughtful observation. We agree that the terminology used to describe the dashboard’s function should be more precise and consistent throughout the manuscript. While the dashboard supports the broader monitoring process, its primary role is to **visualise** compliance results and trends derived from audit data, rather than to monitor the full spectrum of implementation activities. In response, we have revised the manuscript to consistently refer to the dashboard as a **visualisation tool** to assist monitoring of the Directive that presents audit outcomes and compliance trends, while acknowledging that monitoring encompasses a wider set of actions undertaken by HWQld and HHSs.

Additionally, the objective includes "to explore its usability for decision-makers"; it appears the study did not fully realise this aim - it would be expected that some interaction with policy makers took place to explore that (if this was the case, there might not be enough details included to explain this aspect).

Thank you for your observation. We acknowledge that the study did not include a focused exploration of usability for decision-makers. As such, we agree that this objective should be removed from the manuscript to ensure alignment between stated aims and reported findings. The revised version reflects this change.

*Paragraph 3, page 4 now reads:*

*“Still, there is sparse evidence on the use of digital dashboards to monitor preventive health policies, particularly in the domain of nutrition and institutional food environments. While platforms such as the Australian Food Environment Dashboard [6], Global Food Environment Dashboard [7], and Food System Dashboard [8] demonstrate the growing use of interactive tools in public health nutrition, they primarily focus on population-level indicators, disease burden, or broader environmental metrics. These examples reflect a broader trend away from static, report-heavy outputs toward dynamic, user-driven platforms that support tailored data exploration - especially valuable for time-poor policymakers. Building on this momentum, we aim to present a case study for developing a visual digital dashboard to assist in monitoring a preventive health policy related to nutrition within Queensland hospitals and health services.~~and to explore its usability for decision makers~~ ”.*
8. It would be helpful to outline how reporting for this policy has been undertaken to date. Page 6 refers to PDF-based reports, but a clearer description of the existing process would add context; for example, who is responsible for analysing the data after collection, preparing the reports, and disseminating them. If available, information on how the proposed dashboard approach compares in terms of streamlining or feasibility would also strengthen the case for its added value.
We did mention in paragraph 1, page 6 that a team at HWQld is responsible to perform subsequent analysis and report compliance data to the Queensland Department of Health and provide feedback to each HHS on their performance and plans for improvement. We have now moved this to paragraph 2 for a clearer explanation.

Paragraph 3, page 6 now reads:

“*Historically, reporting for the Healthier Food and Drinks at Healthcare Facilities Directive has relied on manual processes, with audit data collected by HHSs. Following data collection, HWQld is responsible for analysing the results, preparing PDF-based summary reports, and disseminating findings to individual HHSs. This process, while effective, has been time-intensive and dependent on static outputs that limit accessibility and real-time engagement. HWQld sought a collaboration with the Queensland Digital Health Centre (The University of Queensland) to explore areas for improvement and expansion in data input, data cleaning and report handling processes.*”

We also clarified in the Results section that the dashboard development process has resulted in faster delivery of food and drinks compliance report to HHSs and Queensland Health as the requirement for HWQ to manually clean and analyse data has been replaced by an automatic process. Paragraph 2, page 11 now reads:

“*The dashboard development process has facilitated faster delivery of the food and drinks compliance report to HHSs and Queensland Health. The requirement for HWQ to manually clean and analyse data has been replaced by an automatic process, this assisted in the ability to send evidence-based outcomes reports to HHSs and Queensland Health in a timely manner.*”
9. Data Preparation and Analysis
The manuscript would benefit from a more detailed description of the data flow between Qualtrics and the dashboard. Key points needing clarification include what data are collected in hospitals (e.g. product photos, nutrition information), how items are classified as green/amber/red (and at what point), who undertakes data cleaning and quality assurance, how discrepancies across services are resolved, and how was data that previously was reported as percentages integrated with the updated reporting on numbers. Greater detail would help readers understand the workflow and effort required to generate dashboard outputs for those wanting to develop their own dashboard.
Thank you for this insightful comment. We agree that a clearer description of the data flow and processing steps would enhance the manuscript’s utility for readers interested in replicating or adapting the dashboard. In response, we have expanded the relevant section to outline the full workflow: from data collection in HHSs via Qualtrics to dashboard integration.

*Paragraphs 1 and 2, page 8 now read:*

*“Prior to 2023, each HHS received a survey towards the end of the calendar year to report by the following January, the proportions of food and drinks from the green, red and amber categories, and artificially sweetened drinks, in their retail outlets and vending machines This process has been in place for several years, and HHSs are familiar with the classification criteria outlined in the Healthier Food and Drinks Directive. In 2023, the survey was modified to report the number of items instead of proportions. This was to reduce the burden on the persons who complete the survey as requiring only simple counts and no calculations. Also introduced in 2023, the outlets needing to report were specified by HWQld to ensure that all retail outlets and vending machine brands within each HHS reported on their compliance within any two-year cycle. This reduced the risk of auditing being limited to the same selected outlets each year, with associated limited visibility of overall outlet performance. The 2023 survey data was captured using Qualtrics, an online survey tool used for teaching, learning, and research. After receiving the survey results back from HHSs, a team at HWQld checked the face validity of the data for each HHS. The data were then extracted into excel files and forwarded to the data analysis team at Queensland Digital Health Centre for processing.*

*Data cleaning and compilation*

*Audit data from each HHS was imported into RStudio (version 4.4.0) for cleaning and manipulation. This process involved checking for data entry errors such as missing values and outliers or inconsistencies. The proportion for each category and outlet compliance was calculated based on the number of items reported for each category. The 2023 dataset was then merged with historical audit data; the resulting cleaned and consolidated dataset was integrated into the digital dashboard.”*
10. Dashboard
The functionality of the dashboard is well outlined, and the weighted outputs by population are a particularly strong feature, demonstrating the potential of a digital approach that integrates data from multiple sources. This aspect is well done.

Thank you for your positive feedback

A few points could be clarified further:
-Figures: In Figure 2, there are no amber results, whereas amber appears in Figure 4; an explanation would be helpful.

Figure 2 presented the proportion of outlets that have green, red, or artificially sweetened drinks that meet specific targets. As outlined in Table S1, there was no specific target set for amber category; therefore, there was no amber in Figure 2. Whereas, in Figure 4, the dashboard displayed the actual percentage of items in each category to observe the longitudinal trend. Clarifications have been added at the footnote of each figure to explain what metrics displayed in each dashboard.

-Flags: More detail is needed on how the dashboard flags specific items, why certain flags were chosen, and how these flags support validity checks, given that most users would not independently verify data quality.

We have specified in the manuscript that additional flags - to identify facilities with large changes and outlets that reported very small or large number of items - were determined pragmatically and based on what is most useful for HWQld – currently the primary user of the dashboard. These functions support the team to assess data validity and initiate follow-up actions.

*Paragraph 1, page 11 now reads:*

*“Figure 5 shows the compliance trend overtime, allowing users to track the changes in overall compliance, as well as averages of food and drinks from the green, red and amber categories and artificially sweetened drinks. In addition to these main pages, we created a flag for large changes (S1 Fig), which helps identify outlets or facilities that had significant changes from the previous year. Outlier flags were also created for outlets reporting very small or large numbers of items (S2 Fig); these thresholds were determined pragmatically, based on what is most useful for HWQld. These flagging functions support the HWQld team -currently the primary user of the dashboard – in assessing data validity and initiate follow-up actions where potential data quality issues are identified.”*

-Communication: It would also be useful to describe how results are (or will be) communicated back to individual retailers (if at all), as this will be important for practical application.
Currently, results are provided to each HHS, along with tailored improvement plans for outlets that did not meet the target. It is the responsibility of each HHS to communicate these findings to their respective outlets. A process is in place to identify retailers that fall within the scope of the Directive and are required to comply with its policy. Future plans include establishing mechanisms to communicate results directly to individual retailers.

*Paragraph 4, page 12 now reads:*

*“The dashboard is currently accessible to policy makers but not available to HHSs. The current design of the dashboard allows for a number of options for sharing; however, maintaining a robust digital dashboard requires significant technical expertise and infrastructure [5]. Ensuring the privacy and security of the data is critical. Effective use of dashboards requires training for users to interpret and use the data correctly [5]. All these aspects need to be considered before sharing the dashboards to broader users. Upcoming efforts will also focus on exploring effective methods for communicating results to individual retailers and determining which entities fall within the scope of the Directive.”*

11. Figures
The mock-up figures illustrating dashboard results are generally self-explanatory, but some features would benefit from additional clarification, for example, through a legend. It is not clear what the 53% value shown for the overall performance metric represents (and for the other, more detailed results), compared to the green/amber/red categories. A greater explanation of how users or policymakers should interpret such metrics would improve clarity, and where the flagging function would be shown.

Thank you for this valuable feedback. We agree that further clarification would enhance the interpretability of the dashboard figures. In response, we have revised the figures and added a legend to interpret visualisations within each dashboard. Additionally, we added two figures in the supplementary material (Figures S1-S2) to indicate where the flagging functions appear.
