# Supplementary material for: Development of a digital tool to assist in monitoring compliance for a public health initiative: “A Better Choice Food and Drink Supply Strategy for Queensland Healthcare Facilities”
Source: PLoS One. 2026 Feb 20;21(2):e0341747. doi: 10.1371/journal.pone.0341747 (PMC12922977; doi:10.1371/journal.pone.0341747)
Supplement: Attachment — Submitted filename: Responses to reviewers.docx [file pone.0341747.s006.docx]

**STATEMENT ADDRESSING REVIEWERS’ COMMENTS**

Note: Responses to reviewers’ comments are in blue. Changes made to the manuscript are in underlined red. Page and paragraph numbers cited herein pertain to the marked-up version provided.

**Reviewer #1:**

Although I am satisfied by the responses to my question, I still feel that you need to correct the editorial and language issues in order to make the manuscript readable. marking of graph axis, their relative size, and the need for each of them need to be evaluated.

Thank you for your comments. We have revised the manuscript, corrected grammar issues, and reviewed the overall readability. We hope this has improved reliability but are happy to address any further specific feedback.

We have also reviewed the figures and appreciate the suggestions regarding axis labelling and relative sizing. However, the visualisations presented in this study were designed for use within an interactive dashboard intended for policy makers and stakeholders. As such, the standards applied to static figures in published materials may not be directly applicable. The dashboard visualisations allow users to interact with the data and access additional detail, which cannot be fully represented in static screenshots included in the manuscript.

**Reviewer #2:**

Thank you very much for addressing all of my previous comments and providing clarifications. I just have a few minor suggestions for further improvement:

1. Abstract - Results section:
The third sentence and the last sentence feel slightly repetitive, and could be reworded for clarity. It might also help to combine the entire visualisation description into one cohesive statement at the end of this section.

The results section of the abstract (on page 3) has been reworded for clarity and now reads:

*“The development process resulted in a replicable digital dashboard for reporting and decision-making. The ABC dashboard provides previous and current compliance data for all HHSs, featuring visualisations that illustrate changes in compliance over time to help identify emerging trends. Users can interact with the dashboard to filter data by HHS, year and by outlet and food or drink type. This digital innovation has facilitated faster delivery of food and drink supply trend analysis and compliance reporting for HHSs and statewide policy makers.”*

2. Abstract - Conclusion:
There is a conflict between singular and plural grammar. Suggest changing to a plural form as it is a conclusion so it refers to more generalisable findings.

Grammar has been checked and changed to plural form. The abstract conclusions section now reads (page 3):

*“Digital dashboards for public health policy compliance enable greater interrogation of data and provide visualisation tools to track trends in compliance over time. This allows more responsive and effective action to increase the impact of public health policies.”*

3. Introduction - Second paragraph:
Briefly explain what 'drilldowns' are as features of the digital tools (e.g., in brackets) for reader clarity.

“Drilldowns” function is now explained in more detailed in paragraph 2 of the Introduction:

*“…Robust infrastructure ensures secure handling of large datasets and timely updates via scalable systems and reliable data pipelines. Intuitive interfaces reduce cognitive load with clean layouts, consistent icons, and interactive features like tooltips and drilldowns (an interactive technique allowing users to see more detailed information when selecting a category).”*

4. Materials and Methods - First paragraph:
In the last sentence, where it says 'AS drinks is no more than 20%', please clarify whether this refers to available drink options only or all options (including food and drink).

Thank you for the suggestion, we have now clarified that the specific target for AS drinks is only for drink options:

*“…The specific target for AS drinks is not more than 20% of all drink options.”*

5. Consistency in terminology:
Throughout the text, there is now some inconsistency in describing the purpose of the digital dashboard (e.g., 'to monitor compliance' vs. 'to visualise compliance').

We acknowledge the inconsistency in how the purpose of the digital dashboard was described. We have revised the manuscript accordingly and now consistently state that the dashboard is designed to **visualise**, rather than monitor, compliance.
